# Shear and Dynamic Compression Modulates the Inflammatory Phenotype of Human Monocytes *in vitro*

**DOI:** 10.3389/fimmu.2019.00383

**Published:** 2019-03-05

**Authors:** Niamh Fahy, Ursula Menzel, Mauro Alini, Martin J. Stoddart

**Affiliations:** AO Research Institute Davos, Davos, Switzerland

**Keywords:** osteoimmunology, immune regulation, fracture repair, mechanoregulation, bone healing, macrophage

## Abstract

Monocytes and their derived macrophages are found at the site of remodeling tissue, such as fracture hematoma, that is exposed to mechanical forces and have been previously implicated in the reparative response. However, the mechanoresponsive of monocytes and macrophages to skeletal tissue-associated mechanical forces and their subsequent contribution to skeletal repair remains unclear. The aim of this study was to investigate the potential of skeletal tissue-associated loading conditions to modulate human monocyte activation and phenotype. Primary human monocytes or the human monocyte reporter cell line, THP1-Blue, were encapsulated in agarose and exposed to a combination of shear and compressive loading for 1 h a day for 3 consecutive days. Exposure of monocytes to mechanical loading conditions increased their pro-inflammatory gene and protein expression. Exposure of undifferentiated monocytes to mechanical loading conditions significantly upregulated gene expression levels of interleukin(IL)-6 and IL-8 compared to free swelling controls. Additionally, multiaxial loading of unstimulated monocytes resulted in increased protein secretion of TNF-α (17.1 ± 8.9 vs. 8 ± 7.4 pg/ml) and MIP-1α (636.8 ± 471.1 vs. 124.1 ± 40.1 pg/ml), as well as IL-13 (42.1 ± 19.8 vs. 21.7 ± 13.6) compared monocytes cultured under free-swelling conditions. This modulatory effect was observed irrespective of previous activation with the M1/pro-inflammatory differentiation stimuli lipopolysaccharide and interferon-γ or the M2/anti-inflammatory differentiation factor interleukin-4. Furthermore, mechanical shear and compression were found to differentially regulate nitric oxide synthase 2 (NOS2) and IL-12B gene expression as well as inflammatory protein production by THP1-Blue monocytes. The findings of this study indicate that human monocytes are responsive to mechanical stimuli, with a modulatory effect of shear and compressive loading observed toward pro-inflammatory mediator production. This may play a role in healing pathways that are mechanically regulated. An in depth understanding of the impact of skeletal tissue-associated mechanical loading on monocyte behavior may identify novel targets to maximize inflammation-mediated repair mechanisms.

## Introduction

The repair process following traumatic injury to the musculoskeletal system is known to be influenced by the mechanical environment. The natural course of bone healing can be intramembranous ossification resulting from stable fracture fixation and subsequent low interfragmentary motion, or endochondral ossification which is associated with moderate interfragmentary movement (1). In addition to driving fracture healing responses, mechanical forces of an appropriate magnitude are also key to maintaining cartilage homeostasis within the articulating joint (2).

A wound healing response is initiated during the process of bone fracture repair, as well as marrow stimulation techniques applied in cartilage repair strategies where the subchondral bone is penetrated. This involves inflammatory cell exudation or infiltration to the site of injury, followed by coagulation activation and fibrin clot formation, which is known to regulate monocyte chemotaxis and proliferation (3, 4). Monocytes and monocyte-derived macrophages are key immune effector cells playing a vital role in host defense, as well as contributing to tissue remodeling and repair (5). Macrophages are associated with a high degree of plasticity having the potential to change phenotype in response to environmental cues, and may be classified according to pro-inflammatory (M1) or anti-inflammatory (M2) subsets (5). Pro-inflammatory macrophages are associated with high production of pro-inflammatory cytokines and increased microbicidal activity, whereas anti-inflammatory macrophages are associated with wound healing and immunoregulatory functions (3, 6).

Infiltrating monocytes and macrophages may influence the success of musculoskeletal tissue repair processes. Macrophage depletion has been previously demonstrated to negatively impact endochondral ossification and subsequently delay bone fracture healing (7–9). Furthermore, monocyte and macrophage associated inflammatory cytokines such as IL-6 and TNF-α are known to promote bone repair, with inhibition of TNF-α signaling shown to delay both intramembranous and endochondral bone formation (10, 11). In contrast to bone healing, pro-inflammatory factors such as IL-1β and TNF-α produced by both activated monocytes and M1 polarized macrophages, induce destructive processes in cartilage tissue including catabolic enzyme expression and reduced extracellular matrix deposition (12–14). However, recruitment of anti-inflammatory macrophages to the site of subchondral drill holes within osteochondral defects using chitosan-glycerol phosphate composites was reported enhance subchondral bone repair and improve cartilage resurfacing, further highlighting the impact of macrophages on skeletal tissue repair (15). Monocytes and macrophages are found at the site of remodeling tissue that is exposed to mechanical forces and have been previously implicated in the reparative response (16–18). As the area of osteoimmunology gains in importance, the influence of mechanical stimulation on immune cell phenotype needs to be investigated in greater detail. However, the responsiveness of macrophages and monocytes, their lineage precursors, to mechanical forces that are native to skeletal tissues and the effect of such mechanical stimuli on macrophage phenotype requires further elucidation. Therefore, the aim of this study was to investigate the impact of mechanical shear and compressive loading on monocyte activation and phenotype. Unstimulated, M1 or M2-stimulated primary human monocytes as well as the human monocyte reporter cell line, THP1-Blue, were exposed to a combination of shear and compressive loading *in vitro*. Gene expression levels of inflammatory mediators and inflammatory protein secretion was assessed following 3 days of mechanical stimulation.

## Materials and Methods

### Human Monocyte Isolation

Human monocytes were isolated from buffy coats left over from voluntary whole blood donations after informed consent of the donors according to the regulations of Swiss Red Cross Blood Service. Buffy coats were processed within 23 h after blood donation by centrifugation at 5,000 g for 15 min and subsequent separation on Compomat G5 (Fresenius, Oberdorf, Switzerland) using top-and-bottom 450 ml blood bag systems pre-filled with Citrate-Dextrose-Phosphate Solution (Fresenius). Buffy coats were anonymized prior to delivery from the Blood Service to AO Institute in line with the ethics code provided by the Swiss Drug Law (Heilmittelgesetz). For the isolation of peripheral blood mononuclear cells (PBMCs), each buffy coat was diluted at a 1:5 ratio with 0.1% bovine serum albumin (BSA) in phosphate buffered saline (PBS). Thirty milliliter of diluted buffy coat was layered on 15 ml of Ficoll and centrifuged at 1,000 g for 15 min without brake. Following centrifugation, the interphase layer containing PBMCs was removed and washed with 0.5% BSA/PBS containing 2 mM EDTA. Isolated PBMCs were labeled with 100 μl of anti-CD14 magnetic bead solution (Miltenyi Biotec Bergisch Gladbach, Germany) in the dark at 4°C for 20 min. Monocytes were isolated utilizing MACS LS Separation columns and a MidiMACS^TM^ Separator (Miltenyi Biotec), according to manufacturer's instructions. Purity of isolated CD14+ cells was assessed by fluorescence activated cell sorting (FACS) analysis. 1 × 10^5^ monocytes were incubated with APC-Cy7-conjugated anti-human CD14 antibody (BD pharmingen) for 20 min in the dark at 4°C. FACS analysis was performed using a BD Aria III machine, and data analyzed using BD FACS Diva 6.1.3 software (BD Biosciences). The average purity of CD14+ monocytes from all donors was found to be 95% (data not shown). Monocytes were isolated from two individual buffy coat donors and pooled per experiment.

### THP1-Blue™ Cell Culture

The human monocyte reporter cell line THP1-Blue^TM^ (InvivoGen, CA, USA) which expresses an NF-κB and AP-1-inducible secreted embryonic alkaline phosphatase (SEAP) reporter gene, was cultured in RPMI-1640 medium (2 mM L-glutamine; Gibco, Carlsbad, USA) supplemented with 1% penicillin/streptomycin (Gibco) and 10% heat inactivated fetal bovine serum (FBS; Pan Biotech, Aidenbach, Germany). Monocyte suspension cultures were maintained at a density of at 3–8 × 100,000 cells/ml.

### Agarose Gel Seeding and Culture

To evaluate the effect of mechanical loading on monocyte phenotype, human primary monocytes and THP1-Blue cells were encapsulated in 2% low melting temperature agarose (Lonza) at a cell density of 3 × 10^6^ monocytes per gel. In brief, a 4% agarose solution was prepared by dissolving low melting temperature agarose in sterile phosphate buffered saline and heated. The 4% agarose solution was cooled and mixed with an equal volume of cells suspended in pre-warmed culture media, composed of RPMI-1640 medium supplemented with 2 mM L-glutamine, 1% penicillin/streptomycin and 10% heat inactivated FBS. Two hundred and fifty microliters of cells/agarose suspension was added to a sterile cap of an Eppendorf tube and gels were allow to set at 37°C for 20 min. All agarose constructs were carefully removed from the Eppendorf cap, placed in a sterile PEEK sample holder and cultured with 2.5 ml of culture medium. To investigate the effect of mechanical loading on macrophage phenotype, CD14+ monocytes were stimulated with 10 ng/ml IFN-γ (PeproTech, Rocky Hill, NJ, USA) and 100 ng/ml lipopolysaccharide to induce differentiation toward a pro-inflammatory/M1 phenotype, 10 ng/ml IL-4 (PeproTech) for an anti-inflammatory/M2 phenotype or unstimulated for 72 h prior to loading. Agarose gels containing THP1-Blue monocytes were prepared 24 h prior to loading.

### Mechanical Loading

CD14+ monocyte seeded agarose gels were mechanically loaded using a custom built multi-axial load bioreactor based on a 32 mm ceramic hip ball that can apply compression, shear or a combination of the two, to the sample as previously described (19, 20). Shear (±25° ball rotation at 1 Hz) and compression (10% compression superimposed on top of a 10% pre-strain at 1 Hz) loading was applied for 1 h a day for 3 consecutive days. This protocol was chosen as it has been shown to direct osteochondral differentiation of human MSCs and therefore we aimed to investigate similar loading patterns on the modulation of macrophage phenotype (21). Control gels were maintained in free-swelling conditions for the duration of the experiment. To investigate the effect of shear or compression alone on monocyte phenotype, THP1-Blue monocytes were stimulated with shear or compression alone as well as multiaxial loading for 1 h a day for 3 consecutive days. Control gels were maintained in free-swelling conditions for the duration of the experiment. Cell culture media was refreshed every 24 h prior to loading.

### Reverse Transcription and PCR

Monocyte-seeded agarose gels were homogenized in 1 ml TRI reagent (Molecular Research Center Inc., Cincinnati, OH, USA). Homogenized samples were supplemented with 100 μl of 1-Bromo-3-chloropropane (Sigma-Aldrich) and processed according to manufacturer's instructions to achieve phase separation. Following phase separation the aqueous phase was removed, supplemented with an equal volume of 70% ethanol (Sigma-Aldrich) and transferred to a RNeasy spin column (Qiagen, Hilden, Germany). RNA was extracted using RNeasy mini spin columns according to manufacturer's instructions. The purity of isolated RNA was assessed using a NanoDrop spectrophotometer (Fisher Scientific, Delaware, USA) based on the absorbance ratios 260/280 nm and 260/230 nm. Reverse transcription was performed using random hexamer primers and TaqMan reverse transcription reagents (Applied Biosystems, Carlsbad, CA, USA). Quantitative real time PCR was performed in 10 μl reactions on cDNA using the Applied Biosystems QuantStudio 6 Flex Real Time PCR system (Applied Biosystems). Primers for cyclooxygenase(COX)-2 (*PTGS2*) were synthesized by Microsynth AG (Balgach, SG, Switzerland; Table 1). Gene expression assays for 18S ribosomal RNA (*18S*), interleukin(IL)-6 (*IL6*), IL-8 (*IL8*), IL-10 (*IL10*), tumor necrosis factor (TNF)-α (*TNF*), chemokine (C-C motif) ligand 18 (*CCL18*), mannose receptor CD206 (*MRC1*), nitric oxide synthase 2 (*NOS2*), and monocyte chemoattractant protein 1 (*CCL2*) were purchased from Applied Biosystems, Switzerland (Table 1). Gene expression levels were normalized to 18S rRNA, and relative expression calculated via a ΔΔCT comparison.

**Table 1:** Gene expression assays utilized for quantitative real time PCR.

**Gene name**	**Alias**	**Assay ID/Primer sequence**
Human 18S rRNA (*18S)*	18S ribosomal RNA (18S rRNA)	Hs99999901_s1
Human interleukin-8 (*IL8)*	C-X-C motif chemokine ligand 8 (CXCL8)	Hs00174103_m1
Human interleukin-10 (*IL10)*	Cytokine Synthesis Inhibitory Factor (CSIF)	Hs00961622_m1
Human interleukin-6 (*IL6)*	B-Cell Stimulatory Factor (BSF)- 2	Hs00985639_m1
Human tumor necrosis factor (*TNF)*	Tumor Necrosis Factor (TNF)-α	Hs01113624_g1
Human chemokine (C-C motif) ligand 18 (*CCL18)*	Macrophage Inflammatory Protein (MIP)-4	Hs00268113_m1
Human mannose receptor, C type 1 (*MRC1)*	Macrophage Mannose Receptor (MMR, CD206)	Hs00267207_m1
Human C-C motif chemokine ligand 2 (*CCL2)*	Monocyte Chemoattractant Protein (MCP)-1	Hs00234140_m1
Human nitric oxide synthase 2 (*NOS2)*	Inducible Nitric Oxide Synthase (INOS)	Hs01075529_m1
Human interleukin 12B (*IL12B)*	Natural Killer Cell Stimulatory Factor	Hs01011518_m1
Human Prostaglandin-Endoperoxide Synthase 2 (*PTGS2)*	Cyclooxygenase (COX)-2	**Forward:** 5′-TTG TAC CCG GAC AGG ATT CTA TG-3′
		**Reverse:** 5′-TGT TTG GAG TGG GTT TCA GAA ATA-3′
		**Probe(5′FAM/3′** **TAMRA):** 5′-GAA AAC TGC TCA ACA CCG GAA TTT TTG ACA A-3′

### Cytokine Assays

Levels of IL-6, IL-8, and CCL18 in cell culture supernatant were quantified utilizing commercially available human IL-6 and CCL18 DuoSet ELISA kits according to manufacturer's instructions (R&D Systems, Minneapolis, Minnesota). Levels of IL-10, IL-13, IL-1β, C-X-C motif chemokine 10 (IP-10), macrophage-derived chemokine (MDC), monocyte chemoattractant protein-1 (MCP-1), macrophage inflammatory protein-1α (MIP-1α), and TNF-α were measured utilizing a Meso Scale Development multiplex assay according to manufacturer's instructions (Meso Scale Discovery, Maryland, USA).

### Secreted Alkaline Phosphatase Assay

Secreted embryonic alkaline phosphatase (SEAP) levels were detected in cell culture supernatant using a QUANTI-Blue^TM^ enzymatic assay (InvivoGen) according to manufacturer's instructions. SEAP levels were determined qualitatively following spectrophotometric measurement at 620 nm.

### Statistical Analysis

IBM SPSS Statistics 21.0 (IBM, New York, USA) and GraphPad Prism software version 6 (GraphPad Software Inc., La Jolla, USA) were used for all statistical analysis. To take donor variability into account between primary monocyte donors, mixed models analysis was applied to test for statistical differences between loaded and free-swelling groups with monocyte donor considered a random factor. THP1-Blue monocyte data sets were analyzed using a Kruskal–Wallis test followed by Dunn's multiple comparisons test. For all analyses, differences were considered statistically significant at *P* < 0.05.

## Results

### Pro-inflammatory Gene and Protein Expression by Differentially Activated Primary Human Monocytes Following Multiaxial Loading

**Figure 1:**
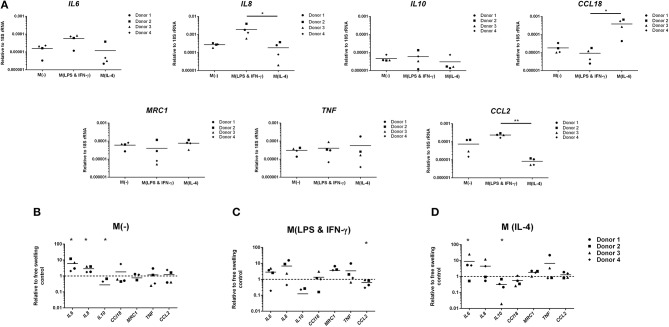
Inflammatory gene expression by differentially activated primary human monocytes following multiaxial loading. **(A)** Gene expression by primary monocytes cultured for 6 days under free-swelling conditions as measured by real-time PCR. Gene expression levels were normalized to the housekeeper gene 18S rRNA. **(B)** Gene expression levels by unstimulated primary human monocytes, or LPS & IFN-γ **(C)** or IL-4 **(D)** stimulated monocytes following 3 days of multiaxial loading. Gene expression levels were normalized to the free swelling control, represented by the dashed line. Data is represented as dot plots including the median for 4 monocyte donors, each assessed in experimental triplicate. Missing points indicate undetectable gene expression. Statistical significance was determined utilizing a mixed model analysis, ^*^P < 0.05.

Monocytes encapsulated in 2% agarose gel were unstimulated, LPS and IFN-γ or IL-4-stimulated for 3 days, prior to subjection to multiaxial loading or free-swelling conditions and analysis of inflammatory gene and protein expression. Monocytes stimulated with LPS and IFN-γ had significantly higher gene expression levels of the pro-inflammatory genes *IL8* and *CCL2* under free-swelling conditions compared to IL-4 stimulated monocytes (10.3- and 28.3-fold increases, respectively), confirming their pro-inflammatory phenotype (Figure 1A). Additionally, IL-4 stimulated monocytes were associated with significantly higher *CCL18* expression compared to LPS and IFN-γ stimulated monocytes (41.5-fold increase), confirming their polarization toward an M2-like phenotype. Unstimulated primary human monocytes significantly upregulated gene expression levels of the pro-inflammatory genes *IL6* (5.9-fold change) and *IL8* (2.8-fold change) following 3 days of mechanical loading compared to monocytes cultured in free-swelling conditions (Figure 1B). Additionally, expression of the anti-inflammatory macrophage marker *IL10* was decreased in all four donors compared to free-swell controls, with gene expression levels undetectable in donors 1 and 3 following loading. No significant difference was observed in the expression levels of inflammatory mediators *CCL18, TNF*, and *CCL2*. Although a similar trend was observed toward inflammatory gene expression by LPS and IFN-γ activated monocytes following mechanical loading, larger variation was observed between donors and these findings were not statistically significant (Figure 1C). However, expression of *CCL2* was significantly decreased (1.9-fold decrease). Additionally, gene expression levels of *IL10* were also undetectable in LPS and IFN-γ stimulated monocytes from donors 1 and 3 following loading. In a similar manner to unstimulated monocytes, IL-4 activated cells were also associated with a significant increase in *IL6* (8.9-fold change) and decrease of *IL10* (3.1-fold) expression (Figure 1D).

**Figure 2:**
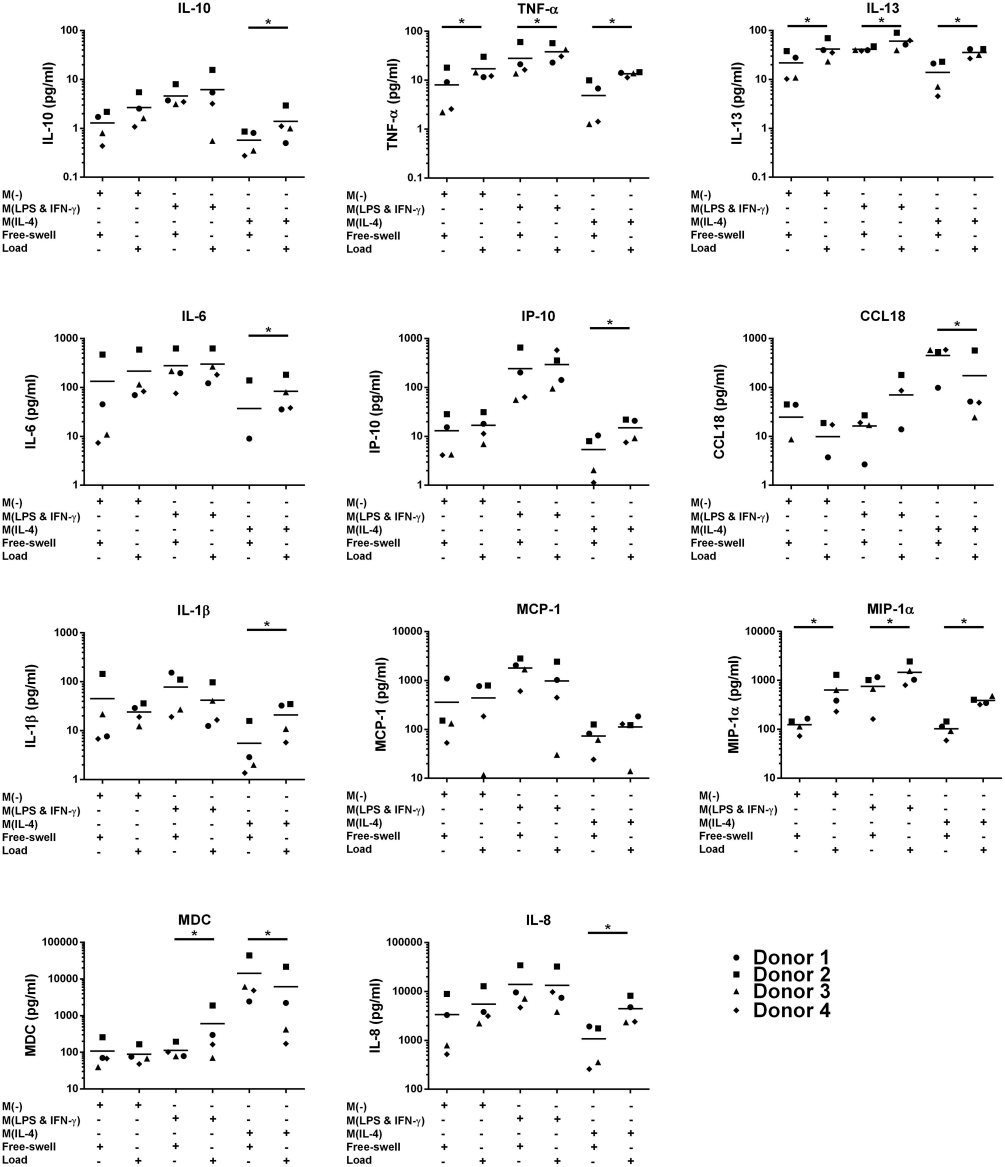
Inflammatory mediator production by primary human monocytes following multiaxial loading. Levels of inflammatory mediators produced by primary monocytes following 3 days of multiaxial loading as quantified by ELISA and multiplex assay. Data is represented as dot plots including the median for 4 monocyte donors, each assessed in experimental triplicate. Missing points indicate undetectable protein levels. Statistical significance was determined utilizing a mixed model analysis, ^*^P < 0.05. IL-6, Interleukin-6; IL-10, Interleukin-10; CCL18, chemokine (C-C motif) ligand 18; TNF-α, Tumor necrosis factor-α; MIP-1α, Macrophage inflammatory protein-1α; IP-10, C-X-C motif chemokine 10; MCP-1, Monocyte chemoattractant protein-1; IL-13, Interleukin-13; MDC, Macrophage-derived chemokine; IL-1β, Interleukin-1β; IL-8, Interleukin-8.

Compared to free-swelling controls, mechanical loading of unstimulated monocytes significantly increased production of the pro-inflammatory mediators TNF-α (17.1 ± 8.9 vs. 8 ± 7.4 pg/ml) and MIP-1α (636.8 ± 471.1 vs. 124.1 ± 40.1 pg/ml), as well as IL-13 (42.1 ± 19.8 vs. 21.7 ± 13.6) (Figure 2). Protein levels of IL-10, CCL18, IP-10, MCP-1, MDC, and IL-1β produced by loaded unstimulated monocytes did not significantly differ from free-swelling controls. In a similar manner to gene expression levels, a trend toward an increase in IL-6 production was observed in response to loading of unstimulated monocytes. However, large donor variation was observed and this finding was not statically significant. Mechanical stimulation of LPS and IFN-γ stimulated monocytes significantly increased MDC levels in addition to TNF-α, MIP-1α, and IL-13 (Figure 2). In a similar manner to gene expression data, IL-4 activated monocytes were associated with significantly increased production of pro-inflammatory factors IL-6, IL-8, TNF-α, MIP-1α, IP-10, IL-13, IL-1β as well as IL-10 in response to mechanical loading, and decreased expression of CCL18 and MDC (Figure 2).

### Inflammatory Gene and Protein Expression by THP1-Blue Monocytes Following Mechanical Shear or Compression

**Figure 3:**
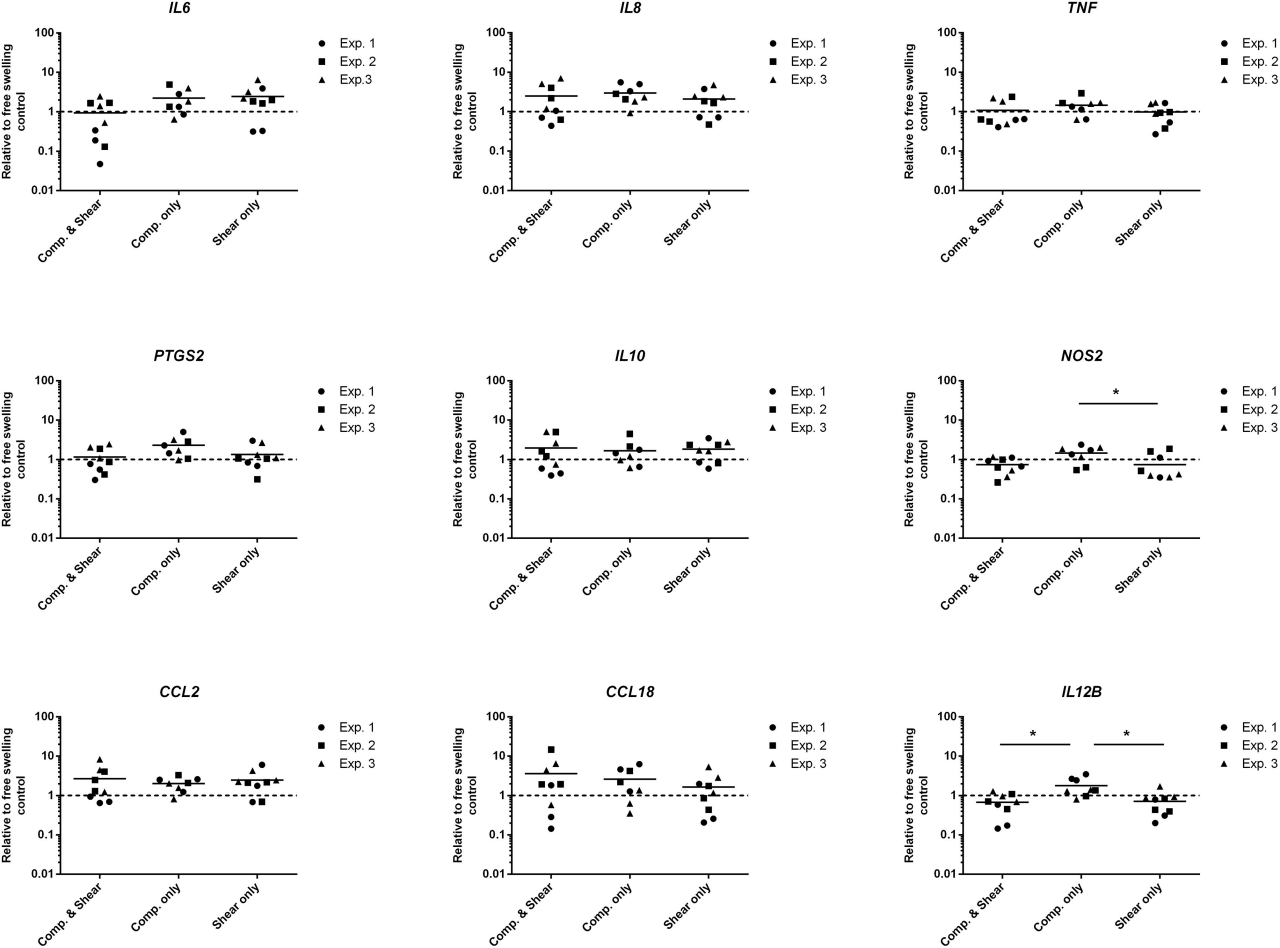
Levels of inflammatory genes expressed by THP1-Blue monocytes in response to multiaxial loading, shear or compression alone. Gene expression by THP1-Blue monocytes following 3 days of multiaxial loading, shear, or compression alone as measured by real-time PCR. Gene expression levels were normalized to the free-swelling control, represented by the dashed line. Data is represented as dot plots including the median for three separate experiments, each assessed in experimental triplicate except for experiment 2 compression only group, which was assessed in duplicate. Statistical significance was determined by a Kruskal–Wallis test followed by Dunn's multiple comparisons test. ^*^P < 0.05. Comp, Compression.

**Figure 4:**
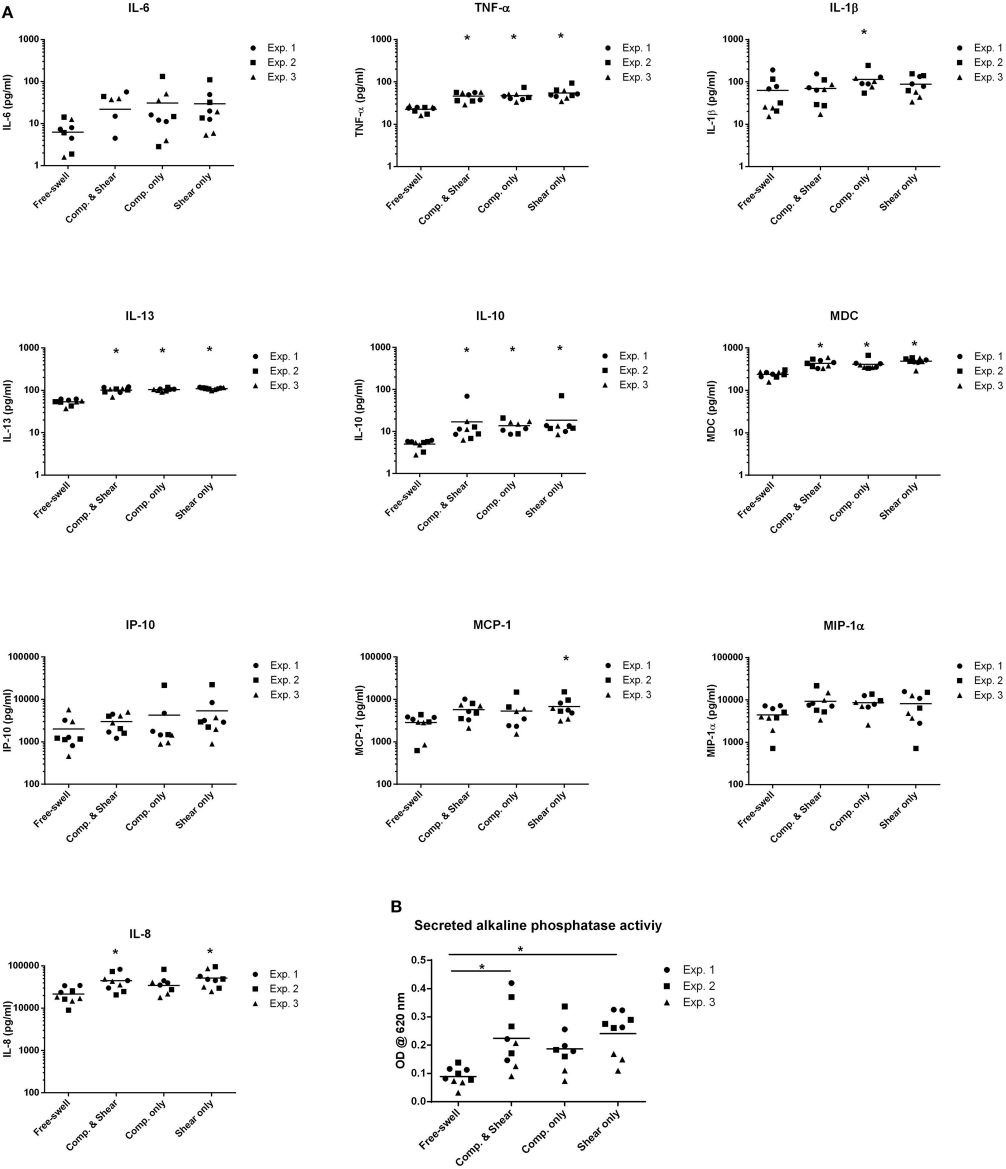
Shear and compression differentially regulate inflammatory mediator expression by THP1-Blue monocytes. **(A)** Levels of inflammatory mediators produced by THP1-Blue monocytes following 3 days of multiaxial loading, shear or compression alone as measured by ELISA and multiplex assay. Protein levels were normalized to the free-swelling control, represented by the dashed line. Data is represented as dot plots including the median for 3 separate experiments, each assessed in experimental triplicate except for experiment 2 compression only group, which was assessed in duplicate. **(B)** SEAP levels detected in cell culture supernatant following 3 days of loading, as measured by spectrophotometric measurement. Statistical significance was determined by a Kruskal–Wallis test followed by Dunn's multiple comparisons test. ^*^P < 0.05. IL-6, Interleukin-6; IL-10, Interleukin-10; CCL18, chemokine (C-C motif) ligand 18; TNF-α, Tumor necrosis factor-α; MIP-1α, Macrophage inflammatory protein-1α; IP-10, C-X-C motif chemokine 10; MCP-1, Monocyte chemoattractant protein-1; IL-13, Interleukin-13; MDC, Macrophage-derived chemokine; IL-1β, Interleukin-1β; IL-8, Interleukin-8; Comp, Compression.

To evaluate the potential of mechanical shear or compression to differentially regulate inflammatory gene and protein expression by human monocytes, unstimulated THP1-Blue monocytes were subjected to multiaxial loading conditions, or mechanical shear or compression alone. THP1-Blue monocytes significantly upregulated gene expression levels of the pro-inflammatory markers *NOS2* and *IL12B* in response to compression alone compared to the combination of compression and shear, as well as shear alone following 3 days of loading (Figure 3). Gene expression levels of *IL6, IL-8, TNF-*α, *PTGS2, IL-10, CCL2*, and *CCL18* did not significantly differ between loading conditions, or compared to free-swelling controls at this time point. However, significantly increased levels of TNF-α, IL-10, IL-8, IL-13, and MDC were detected in the cell culture media harvested from gels subjected to the combination of compression and shear, as well as compression and shear alone for 3 days compared to control (Figure 4A). Additionally, the application of compressive loading alone significantly upregulated IL-1β production by monocytes, whereas shear alone increased the release of MCP-1 compared to all other culture conditions. Levels of IL-6 and IP-10 did not significantly differ in response to any loading condition compared to free-swelling cultures. In addition to modulating inflammatory cytokine production, the application of both compression and shear or shear alone induced the release of secreted alkaline phosphatase by THP1-Blue monocytes, indicative of NF-κB and AP-1 transcription factor activation (Figure 4B).

## Discussion

Monocytes and their derived macrophages are considered key players in tissue remodeling and repair processes. Mechanical loading has been previously shown to influence the levels or pro and anti-inflammatory macrophages in a model of tendon healing (18). Additionally, cyclic strain has been reported to modulate macrophage polarization state toward a reparative phenotype which promoted extracellular matrix synthesis (22). Monocytes are found at the site of skeletal tissue injuries resulting from either traumatic bone fractures, or microdrilling of the subchondral bone to facilitate microfracture-mediated cartilage repair (23). However, the mechanoresponsive of monocytes and their derived macrophages to skeletal tissue-associated mechanical forces, and their subsequent contribution to skeletal repair remains unclear. The aim of this study was to investigate the potential of shear and compressive forces to modulate human monocyte activation and phenotype. In the present study, exposure of monocytes to mechanical loading conditions increased their production of pro-inflammatory mediators. Furthermore, mechanical loading modulated the production of inflammatory factors produced by monocytes irrespective of previous activation with the M1/pro-inflammatory differentiation stimuli LPS and IFN-γ or the M2/anti-inflammatory differentiation factor IL-4.

Bone fractures associated with less mechanical stability are known to heal via the process of endochondral ossification, involving inflammation, callus formation and tissue remodeling processes (1). Infiltration of macrophages into the fracture callus occurs at an early stage of fracture healing, and inhibition of macrophage recruitment impairs vascularization, decreases callus formation and delays repair (24). Macrophages are associated with a high degree of plasticity and can change phenotype according to environmental cues, encompassing both pro-inflammatory/M1 and anti-inflammatory/M2 phenotypes (5). In an experimental osteotomy model, M1-polarized macrophages were identified as the primary macrophage phenotype in the osteotomy area 24 h post-surgery (7). Interestingly, M1 polarized macrophages have also been reported to promote the osteogenic differentiation of bone marrow-derived mesenchymal stem cells (MSCs) (25). Furthermore, macrophage infiltration and prevalence of a M1-like phenotype has been observed in association with MSC-mediated bone repair *in vivo* (26, 27). In the present study, we have observed a skewing of monocyte activation toward a M1-like phenotype following 3 days of shear and compressive loading, highlighting the responsiveness of human monocytes to mechanical stimuli. These findings may shed some light on how the biomechanical environment may play a role in guiding monocyte/macrophage polarization, and potentially contribute to skeletal tissue repair.

In the present study, we have observed an increase in TNF-α, MIP-1α, and IL-13 protein production by unstimulated as well as LPS and IFN-γ and IL-4 activated monocytes following mechanical shear and compression. Two of four donors in the unstimulated group also substantially increased IL-6 production upon loading. Furthermore, levels of the pro-inflammatory cytokines IL-1β, IL-8, and IL-6 produced by IL-4 activated monocytes were increased following loading. Additionally, gene expression levels of IL-8 and IL-6 were increased by unstimulated primary human monocytes subjected to the combination of mechanical compression and shear. This could indicate that these factors would be induced within an unstable fracture. Previous reports have highlighted an influence of mechanical stimuli resulting from fracture fixation stability upon gene expression of matrix metalloproteinase (MMP)-9 and MMP-13 by fracture hematoma in rats (28). Both MMP-9 and MMP-13 are known to play a key role during the process of endochondral bone formation, facilitating extracellular matrix and cell migration (29). Additional studies have demonstrated an upregulation in the expression of genes involved in cartilage and skeletal development by callus tissue following mechanical stimulation, in a rat osteotomy model (30). However, the impact of mechanical stimuli upon the induction of inflammatory gene expression by fracture hematoma *in vivo* requires further investigation. Production of IL-6 and TNF-α is characteristic of activated monocytes and pro-inflammatory M1 macrophages (5, 31). Both IL-6 and TNF-α signaling are known to play a key role in bone fracture healing (32, 33). Additionally, TNF-α is involved in osteoclastic bone resorption (34). Interestingly, in addition to acting as a chemotactic cytokine for monocytes and neutrophils, IL-8 has been reported by Ringe et al. to induce migration of human MSCs (35, 36). Furthermore, IL-8 is known to promote angiogenesis (37, 38). Mechanical stimulation of early human fracture hematoma has also been previously reported to result in increased production of the pro-angiogenic protein vascular endothelial growth factor (VEGF) (39). MIP-1α, also known as CCL3, has been previously implicated in the recruitment of macrophages to the site of injury during bone repair (40). In contrast to the observed upregulation of pro-inflammatory mediators by monocytes in response to mechanical loading, we also detected increased levels of IL-13 protein irrespective of cell activation with LPS and IFN-γ or IL-4. The pleiotropic cytokine IL-13 is known to polarize macrophages toward an M2 phenotype, encompassing anti-inflammatory and tissue repair subsets (41). Additionally, IL-13 is a key mediator of tissue fibrosis, reported to stimulate transforming growth factor (TGF)-β1 production by monocytes and macrophages, as well as increasing TGF-β1 activation (42, 43). TGF-β signaling may promote extracellular matrix deposition and tissue remodeling (44, 45). In addition to mediating tissue fibrosis and macrophage polarization, a role for IL-13 in osteoclast differentiation and bone resorption has been previously highlighted (46). Macrophages have been previously reported to change their phenotype throughout the course of bone healing, with a more predominant role of the M2 subset identified at later stages of repair (7). Given that in the current study loading of monocytes also resulted in production of the M2-polarization factor IL-13, whether a longer duration of loading may switch the balance from M1/M2 requires further investigation.

Having identified an influence of mechanical loading upon the phenotype of M1 or M2-differentiated as well as undifferentiated primary human monocytes, we next sought to investigate whether shear forces or compression alone may be responsible for this effect. In addition to compressive loading, cartilage in the articulating joint and fractures that have not been rigidly fixated, are also subjected to shear. Therefore, we next sought to examine whether shear or compression alone may exert differential effects on undifferentiated monocytes, to gain further insight into whether loading associated with various skeletal tissues may differentially modulate monocyte activation. The human monocyte cell line THP1-Blue^TM^ was utilized to assess the effect of shear, compression or the combination of both on inflammatory mediator expression by monocytes. THP1-Blue^TM^ cells are a reporter cell line, which express secreted embryonic alkaline phosphatase (SEAP) following activation of the transcription factors NF-κB and AP-1. Both NF-κB and AP-1 are activated in monocytes following toll-like receptor 4 (TLR4) stimulation and are involved in the induction of inflammatory gene expression (47). In a similar manner to primary human monocytes, the application of both compression and shear increased expression of inflammatory mediators TNF-α, IL-13, macrophage-derived chemokine (MDC), and IL-10. Interestingly, IL-10 is an anti-inflammatory cytokine but is also produced by monocytes in response to pro-inflammatory stimulation a part of a regulatory feedback mechanism (48). Furthermore, IL-10 is a factor also known to induce the differentiation of macrophages toward an anti-inflammatory phenotype (5). MDC is chemotactic for monocytes and is also considered a marker of M2 macrophages (49, 50). Additionally, we observed differential effects of compression or shear alone on monocyte phenotype. Application of compression alone increased expression of the pro-inflammatory genes *IL12B* and *NOS2* compared to shear alone or the combination of both stimuli. Interestingly, inducible nitric oxide synthase, which is encoded by the gene *NOS2*, has been previously shown to be expressed the initial phase of bone fracture repair (51). Compression alone also significantly increased IL-1β production compared to control, whereas shear alone was found to increase MCP-1. Furthermore, stimulation with both compression and shear or shear alone significantly increased SEAP expression compared to free-swelling controls, suggestive of potential TLR4 activation by monocytes in response to shear force (47). TLR4 has been previously implicated in the pro-inflammatory response of chondrocytes to high fluid shear, and increasing evidence highlights a role of TLR4 activation in inflammatory and catabolic processes associated with osteoarthritis pathogenesis (52–54). Our present findings may provide further insight to the mechanism of the effect of mechanical loading on monocyte activation, however further investigation is required to evaluate the effect of such mechanical stimuli directly on monocyte TLR expression and activation. These mechanically induced changes suggest that the initial monocyte containing hematoma would respond to mechanical motion by upregulating pro-inflammatory cytokines. This could be a danger signal that recruits cells to the site of damage and regulates their response. Rigid fixation would reduce this inflammatory signal leading to a different response. However, further investigation is required to fully determine the impact of mechanical stimuli resulting from fracture fixation stability upon monocyte behavior *in vivo*, and the subsequent influence of mechanically-stimulated monocytes upon skeletal tissue repair.

The findings of the present study highlight the mechanical sensitivity of human monocytes to skeletal tissue-associated loading conditions. Monocyte-derived macrophages have been previously shown to respond to mechanical strain *in vitro*, with an observed upregulation of MMP-1 and MMP-3 expression as well as the transcription factors c-fos and c-jun (55). Furthermore, Yang et al. highlighted a potential role of mechanical strain in the induction of monocyte to macrophage differentiation, mediated by upregulation of the monocyte differentiation-associated transcription factor PU.1 (55). In line with our findings, shear stress has also been shown to promote macrophage differentiation toward a pro-inflammatory M1-like phenotype in a model of atherosclerosis (56). Interestingly, extracellular physical cues resulting from surface stiffness have been reported to modulate TLR signaling by macrophages (57). However, whether these signaling pathways play a role in the responsiveness of monocytes and monocyte-derived macrophages to shear and compressive forces native to skeletal tissue requires further elucidation. This study has several limitations. Primary peripheral blood monocytes treated with LPS and IFN-γ or IL-4 were used as a model *in vitro* culture system to evaluate the effect of loading on M1 or M2-polarized cells, respectively. Investigation of the effect of mechanical loading on M1 and M2 pre-differentiated macrophages and a longer duration of study may be required to specifically examine the modulatory effect of mechanical loading on macrophage polarization state. Additionally, 2% agarose was used in this study as a cell-carrier system in our *in vitro* model to investigate the short-term response of human monocytes to skeletal tissue-associated mechanical stimuli. However, previous studies have highlighted an impact of different scaffold materials toward the cellular mechanical response (58). Therefore, further investigation may be required to determine whether monocyte interactions with different scaffold materials such as fibrin gels, as a more specific model of the wound healing phase of tissue repair, may determine their response to such mechanical stimuli. Furthermore, additional examination utilizing *in vivo* models of fracture healing is required to relate this observed induction of inflammatory mediators by mechanical loaded monocytes to skeletal tissue repair.

In conclusion, the findings of the present study indicate that human monocytes are responsive to mechanical stimuli, with a modulatory effect of shear and compressive loading observed toward pro-inflammatory mediator production. An in depth understanding of the impact of skeletal tissue-associated mechanical loading on monocyte behavior and their subsequent influence on local cellular responses and tissue repair processes, may identify novel strategies to maximize inflammation-mediated repair mechanisms. Furthermore, the findings of this study may provide insights for the development of novel rehabilitation medicine strategies to improve therapeutic outcome for skeletal tissue repair.

## Data Availability

The datasets generated for this study are available on request to the corresponding author.

## Author Contributions

NF performed the experiments, processed samples, analyzed, and interpreted the data. UM processed samples and analyzed the data. MA supported in data analysis and interpretation. MS conceived and designed the study, and interpreted the data. NF, UM, MA, and MS drafted and critically revised the manuscript for important intellectual content. All authors have approved the final submitted manuscript.

### Conflict of Interest Statement

The authors declare that the research was conducted in the absence of any commercial or financial relationships that could be construed as a potential conflict of interest.

## References

[1] Claes L Recknagel S Ignatius A. Fracture healing under healthy and inflammatory conditions. Nat Rev Rheumatol. (2012) 8:133–43. 10.1038/nrrheum.2012.1 22293759

[2] Sanchez-Adams J Leddy HA McNulty AL O'Conor CJ Guilak F. The mechanobiology of articular cartilage: bearing the burden of osteoarthritis. Curr Rheumatol Rep. (2014) 16:451. 10.1007/s11926-014-0451-6 25182679 PMC4682660

[3] Johnson K Aarden L Choi Y De Groot E Creasey A. The proinflammatory cytokine response to coagulation and endotoxin in whole blood. Blood. (1996) 87:5051–60. 8652818

[4] Altieri DC. Proteases and protease receptors in modulation of leukocyte effector functions. J Leukocyte Biol. (1995) 58:120–7. 10.1002/jlb.58.2.120 7643007

[5] Mosser DM Edwards JP. Exploring the full spectrum of macrophage activation. Nat Rev Immunol. (2008) 8:958–69. 10.1038/nri2448 19029990 PMC2724991

[6] Murray PJ Allen JE Biswas SK Fisher EA Gilroy DW Goerdt S. Macrophage activation and polarization: nomenclature and experimental guidelines. Immunity. (2014) 41:14–20. 10.1016/j.immuni.2014.06.008 25035950 PMC4123412

[7] Schlundt C El Khassawna T Serra A Dienelt A Wendler S Schell H. Macrophages in bone fracture healing: their essential role in endochondral ossification. Bone. (2018) 106:78–89. 10.1016/j.bone.2015.10.019 26529389

[8] Raggatt LJ Wullschleger ME Alexander KA Wu AC Millard SM Kaur S. Fracture healing via periosteal callus formation requires macrophages for both initiation and progression of early endochondral ossification. Am J Pathol. (2014) 184:3192–204. 10.1016/j.ajpath.2014.08.017 25285719

[9] Vi L Baht GS Whetstone H Ng A Wei Q Poon R. Macrophages promote osteoblastic differentiation *in-vivo*: implications in fracture repair and bone homeostasis. J Bone Miner Res. (2015) 30:1090–102. 10.1002/jbmr.2422 25487241

[10] Gerstenfeld LC Cullinane DM Barnes GL Graves DT Einhorn TA. Fracture healing as a post-natal developmental process: molecular, spatial, and temporal aspects of its regulation. J Cell Biochem. (2003) 88:873–84. 10.1002/jcb.10435 12616527

[11] Gerstenfeld LC Cho TJ Kon T Aizawa T Cruceta J Graves BD. Impaired intramembranous bone formation during bone repair in the absence of tumor necrosis factor-alpha signaling. Cells Tissues Organs. (2001) 169:285–94. 10.1159/000047893 11455125

[12] Westacott CI Barakat AF Wood L Perry MJ Neison P Bisbinas I. Tumor necrosis factor alpha can contribute to focal loss of cartilage in osteoarthritis. Osteoarthr Cartil. (2000) 8:213–21. 10.1053/joca.1999.0292 10806049

[13] Shakibaei M John T Schulze-Tanzil G Lehmann I Mobasheri A. Suppression of NF-kappaB activation by curcumin leads to inhibition of expression of cyclo-oxygenase-2 and matrix metalloproteinase-9 in human articular chondrocytes: implications for the treatment of osteoarthritis. Biochem Pharmacol. (2007) 73:1434–45. 10.1016/j.bcp.2007.01.005 17291458

[14] Johnson K Hashimoto S Lotz M Pritzker K Terkeltaub R. Interleukin-1 induces pro-mineralizing activity of cartilage tissue transglutaminase and factor XIIIa. Am J Pathol. (2001) 159:149–63. 10.1016/S0002-9440(10)61682-3 11438463 PMC1850418

[15] Hoemann CD Chen G Marchand C Tran-Khanh N Thibault M Chevrier A. Scaffold-guided subchondral bone repair: implication of neutrophils and alternatively activated arginase-1+ macrophages. Am J Sports Med. (2010) 38:1845–56. 10.1177/0363546510369547 20522834

[16] Hibino N Yi T Duncan DR Rathore A Dean E Naito Y. A critical role for macrophages in neovessel formation and the development of stenosis in tissue-engineered vascular grafts. FASEB J. (2011) 25:4253–63. 10.1096/fj.11-186585 21865316 PMC3236622

[17] Arras M Ito WD Scholz D Winkler B Schaper J Schaper W. Monocyte activation in angiogenesis and collateral growth in the rabbit hindlimb. J Clin Invest. (1998) 101:40–50. 10.1172/JCI119877 9421464 PMC508538

[18] Blomgran P Blomgran R Ernerudh J Aspenberg P. A possible link between loading, inflammation and healing: immune cell populations during tendon healing in the rat. Sci Rep. (2016) 6:29824. 10.1038/srep29824 27405922 PMC4942825

[19] Wimmer MA Grad S Kaup T Hanni M Schneider E Gogolewski S. Tribology approach to the engineering and study of articular cartilage. Tissue Eng. (2004) 10:1436–45. 10.1089/ten.2004.10.1436 15588403

[20] Neumann AJ Gardner OF Williams R Alini M Archer CW Stoddart MJ. Human articular cartilage progenitor cells are responsive to mechanical stimulation and adenoviral-mediated overexpression of bone-morphogenetic protein 2. PLoS ONE. (2015) 10:e0136229. 10.1371/journal.pone.0136229 26292283 PMC4546341

[21] Li Z Kupcsik L Yao SJ Alini M Stoddart MJ. Mechanical load modulates chondrogenesis of human mesenchymal stem cells through the TGF-beta pathway. J Cell Mol Med. (2010) 14:1338–46. 10.1111/j.1582-4934.2009.00780.x 19432813 PMC3828850

[22] Ballotta V Driessen-Mol A Bouten CV Baaijens FP. Strain-dependent modulation of macrophage polarization within scaffolds. Biomaterials. (2014) 35:4919–28. 10.1016/j.biomaterials.2014.03.002 24661551

[23] Hoff P Gaber T Strehl C Schmidt-Bleek K Lang A Huscher D. Immunological characterization of the early human fracture hematoma. Immunol Res. (2016) 64:1195–206. 10.1007/s12026-016-8868-9 27629117

[24] Xing Z Lu C Hu D Yu YY Wang X Colnot C. Multiple roles for CCR2 during fracture healing. Dis Models Mech. (2010) 3:451–8. 10.1242/dmm.003186 20354109 PMC2898536

[25] Guihard P Danger Y Brounais B David E Brion R Delecrin J. Induction of osteogenesis in mesenchymal stem cells by activated monocytes/macrophages depends on oncostatin M signaling. Stem Cells. (2012) 30:762–72. 10.1002/stem.1040 22267310

[26] Tour G Wendel M Tcacencu I. Bone marrow stromal cells enhance the osteogenic properties of hydroxyapatite scaffolds by modulating the foreign body reaction. J Tissue Eng Regen Med. (2014) 8:841–9. 10.1002/term.1574 22782939

[27] Gamblin AL Brennan MA Renaud A Yagita H Lezot F Heymann D. Bone tissue formation with human mesenchymal stem cells and biphasic calcium phosphate ceramics: the local implication of osteoclasts and macrophages. Biomaterials. (2014) 35:9660–7. 10.1016/j.biomaterials.2014.08.018 25176068

[28] Ode A Duda GN Geissler S Pauly S Ode JE Perka C. Interaction of age and mechanical stability on bone defect healing: an early transcriptional analysis of fracture hematoma in rat. PLoS ONE. (2014) 9:e106462. 10.1371/journal.pone.0106462 25187955 PMC4154721

[29] Ortega N Behonick D Stickens D Werb Z. How proteases regulate bone morphogenesis. Ann N Y Acad Sci. (2003) 995:109–16. 10.1111/j.1749-6632.2003.tb03214.x 12814943

[30] Salisbury Palomares KT Gerstenfeld LC Wigner NA Lenburg ME Einhorn TA Morgan EF Transcriptional profiling and biochemical analysis of mechanically induced cartilaginous tissues in a rat model. Arthritis Rheum. (2010) 62:1108–18. 10.1002/art.27343 20131271 PMC2929815

[31] Agbanoma G Li C Ennis D Palfreeman AC Williams LM Brennan FM. Production of TNF-alpha in macrophages activated by T cells, compared with lipopolysaccharide, uses distinct IL-10-dependent regulatory mechanism. J Immunol. (2012) 188:1307–17. 10.4049/jimmunol.1100625 22219323

[32] Gerstenfeld LC Cho TJ Kon T Aizawa T Tsay A Fitch J. Impaired fracture healing in the absence of TNF-alpha signaling: the role of TNF-alpha in endochondral cartilage resorption. J Bone Miner Res. (2003) 18:1584–92. 10.1359/jbmr.2003.18.9.1584 12968667

[33] Yang X Ricciardi BF Hernandez-Soria A Shi Y Pleshko Camacho N Bostrom MP. Callus mineralization and maturation are delayed during fracture healing in interleukin-6 knockout mice. Bone. (2007) 41:928–36. 10.1016/j.bone.2007.07.022 17921078 PMC2673922

[34] Bertolini DR Nedwin GE Bringman TS Smith DD Mundy GR. Stimulation of bone resorption and inhibition of bone formation *in vitro* by human tumour necrosis factors. Nature. (1986) 319:516–8. 10.1038/319516a0 3511389

[35] Mukaida N. Pathophysiological roles of interleukin-8/CXCL8 in pulmonary diseases. Am J Physiol Lung Cell Mol Physiol. (2003) 284:L566–77. 10.1152/ajplung.00233.2002 12618418

[36] Ringe J Strassburg S Neumann K Endres M Notter M Burmester GR Towards *in situ* tissue repair: human mesenchymal stem cells express chemokine receptors CXCR1, CXCR2 and CCR2, and migrate upon stimulation with CXCL8 but not CCL2. J Cell Biochem. (2007) 101:135–46. 10.1002/jcb.21172 17295203

[37] Brat DJ Bellail AC Van Meir EG. The role of interleukin-8 and its receptors in gliomagenesis and tumoral angiogenesis. Neuro Oncol. (2005) 7:122–33. 10.1215/S1152851704001061 15831231 PMC1871893

[38] Li A Dubey S Varney ML Dave BJ Singh RK. IL-8 directly enhanced endothelial cell survival, proliferation, and matrix metalloproteinases production and regulated angiogenesis. J Immunol. (2003) 170:3369–76. 10.4049/jimmunol.170.6.3369 12626597

[39] Groothuis A Duda GN Wilson CJ Thompson MS Hunter MR Simon P. Mechanical stimulation of the pro-angiogenic capacity of human fracture haematoma: involvement of VEGF mechano-regulation. Bone. (2010) 47:438–44. 10.1016/j.bone.2010.05.026 20580871

[40] Kawao N Tamura Y Horiuchi Y Okumoto K Yano M Okada K. The tissue fibrinolytic system contributes to the induction of macrophage function and CCL3 during bone repair in mice. PLoS ONE. (2015) 10:e0123982. 10.1371/journal.pone.0123982 25893677 PMC4404328

[41] Van Dyken SJ Locksley RM Interleukin-4- and interleukin-13-mediated alternatively activated macrophages: roles in homeostasis and disease. Ann Rev Immunol. (2013) 31:317–43. 10.1146/annurev-immunol-032712-095906 23298208 PMC3606684

[42] Fichtner-Feigl S Strober W Kawakami K Puri RK Kitani A. IL-13 signaling through the IL-13alpha2 receptor is involved in induction of TGF-beta1 production and fibrosis. Nat Med. (2006) 12:99–106. 10.1038/nm1332 16327802

[43] Lee CG Homer RJ Zhu Z Lanone S Wang X Koteliansky V. Interleukin-13 induces tissue fibrosis by selectively stimulating and activating transforming growth factor beta(1). J Exp Med. (2001) 194:809–21. 10.1084/jem.194.6.809 11560996 PMC2195954

[44] Burns WC Twigg SM Forbes JM Pete J Tikellis C Thallas-Bonke V. Connective tissue growth factor plays an important role in advanced glycation end product-induced tubular epithelial-to-mesenchymal transition: implications for diabetic renal disease. J Am Soc Nephrol. (2006) 17:2484–94. 10.1681/ASN.2006050525 16914537

[45] Zhu Z Homer RJ Wang Z Chen Q Geba GP Wang J. Pulmonary expression of interleukin-13 causes inflammation, mucus hypersecretion, subepithelial fibrosis, physiologic abnormalities, and eotaxin production. J Clin Invest. (1999) 103:779–88. 10.1172/JCI5909 10079098 PMC408149

[46] Palmqvist P Lundberg P Persson E Johansson A Lundgren I Lie A. Inhibition of hormone and cytokine-stimulated osteoclastogenesis and bone resorption by interleukin-4 and interleukin-13 is associated with increased osteoprotegerin and decreased RANKL and RANK in a STAT6-dependent pathway. J Biol Chem. (2006) 281:2414–29. 10.1074/jbc.M510160200 16251181

[47] Guha M Mackman N. LPS induction of gene expression in human monocytes. Cell Signal. (2001) 13:85–94. 10.1016/S0898-6568(00)00149-2 11257452

[48] Donnelly RP Freeman SL Hayes MP. Inhibition of IL-10 expression by IFN-gamma up-regulates transcription of TNF-alpha in human monocytes. J Immunol. (1995) 155:1420–7. 7636207

[49] Godiska R Chantry D Raport CJ Sozzani S Allavena P Leviten D. Human macrophage-derived chemokine (MDC), a novel chemoattractant for monocytes, monocyte-derived dendritic cells, and natural killer cells. J Exp Med. (1997) 185:1595–604. 10.1084/jem.185.9.1595 9151897 PMC2196293

[50] Jaguin M Houlbert N Fardel O Lecureur V. Polarization profiles of human M-CSF-generated macrophages and comparison of M1-markers in classically activated macrophages from GM-CSF and M-CSF origin. Cell Immunol. (2013) 281:51–61. 10.1016/j.cellimm.2013.01.010 23454681

[51] Zhu W Diwan AD Lin JH Murrell GA. Nitric oxide synthase isoforms during fracture healing. J Bone Miner Res. (2001) 16:535–40. 10.1359/jbmr.2001.16.3.535 11277271

[52] Wang P Zhu F Tong Z Konstantopoulos K. Response of chondrocytes to shear stress: antagonistic effects of the binding partners Toll-like receptor 4 and caveolin-1. FASEB J. (2011) 25:3401–15. 10.1096/fj.11-184861 21715681 PMC3177565

[53] Kim HA Cho ML Choi HY Yoon CS Jhun JY Oh HJ. The catabolic pathway mediated by Toll-like receptors in human osteoarthritic chondrocytes. Arthritis Rheum. (2006) 54:2152–63. 10.1002/art.21951 16802353

[54] Bobacz K Sunk IG Hofstaetter JG Amoyo L Toma CD Akira S. Toll-like receptors and chondrocytes: the lipopolysaccharide-induced decrease in cartilage matrix synthesis is dependent on the presence of toll-like receptor 4 and antagonized by bone morphogenetic protein 7. Arthritis Rheum. (2007) 56:1880–93. 10.1002/art.22637 17530716

[55] Yang JH Sakamoto H Xu EC Lee RT. Biomechanical regulation of human monocyte/macrophage molecular function. Am J Pathol. (2000) 156:1797–804. 10.1016/S0002-9440(10)65051-1 10793091 PMC1876939

[56] Seneviratne AN Cole JE Goddard ME Park I Mohri Z Sansom S. Low shear stress induces M1 macrophage polarization in murine thin-cap atherosclerotic plaques. J Mol Cell Cardiol. (2015) 89(Pt B):168–72. 10.1016/j.yjmcc.2015.10.034 26523517

[57] Gruber E Heyward C Cameron J Leifer C. Toll-like receptor signaling in macrophages is regulated by extracellular substrate stiffness and Rho-associated coiled-coil kinase (ROCK1/2). Int Immunol. (2018) 30:267–78. 10.1093/intimm/dxy027 29800294 PMC5967458

[58] Hunter CJ Mouw JK Levenston ME. Dynamic compression of chondrocyte-seeded fibrin gels: effects on matrix accumulation and mechanical stiffness. Osteoarthr Cartil. (2004) 12:117–30. 10.1016/j.joca.2003.08.009 14723871

